# 7′-(Naphthalen-1-yl)-5′′-[(naphthalen-2-yl)­methyl­idene]-1′,3′,5′,6′,7′,7a′-hexa­hydro­dispiro­[acenaphthene-1,5′-pyrrolo­[1,2-*c*]thia­zole-6′,3′′-piperidine]-2(1*H*),4′′-dione

**DOI:** 10.1107/S1600536812007271

**Published:** 2012-02-29

**Authors:** J. Suresh, R. Vishnupriya, R. Ranjith Kumar, S. Sivakumar, P. L. Nilantha Lakshman

**Affiliations:** a Department of Physics, The Madura College, Madurai 625 011, India; b Department of Organic Chemistry, School of Chemistry, Madurai Kamaraj University, Madurai 625 021, India; c Department of Food Science and Technology, University of Ruhuna, Mapalana, Kamburupitiya 81100, Sri Lanka

## Abstract

In the title compound, C_43_H_34_N_2_O_2_S, the six-membered piperidine ring adopts a half-chair conformation. The five-membered thia­zole ring adopts a slightly twisted envelope conformation and the pyrrole ring adopts an envelope conformation; in each case, the C atom linking the rings is the flap atom. The mol­ecular structure features inter- and intra­molecular C—H⋯O inter­actions. Furthermore, the crystal packing is stabilized by four inter­molecular C—H⋯π inter­actions.

## Related literature

For hydrogen bonding, see: Bernstein *et al.* (1995 ▶). For the importance of spiro compounds, see: Kobayashi *et al.* (1991 ▶); James *et al.* (1991 ▶); Caramella & Grunanger (1984 ▶).
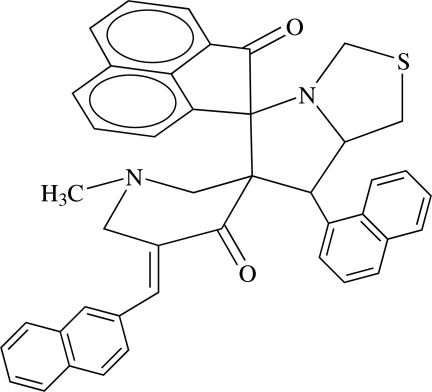


## Experimental

### 

#### Crystal data

C_43_H_34_N_2_O_2_S
*M* *_r_* = 642.78
Monoclinic, 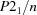

*a* = 15.8335 (4) Å
*b* = 9.2147 (2) Å
*c* = 23.9951 (5) Å
β = 109.103 (1)°
*V* = 3308.12 (13) Å^3^
*Z* = 4
Mo *K*α radiation
μ = 0.14 mm^−1^
*T* = 293 K
0.21 × 0.17 × 0.12 mm

#### Data collection

Bruker Kappa APEXII diffractometer
Absorption correction: multi-scan (*SADABS*; Sheldrick, 1996 ▶) *T*_min_ = 0.967, *T*_max_ = 0.974
40746 measured reflections
9096 independent reflections
5614 reflections with *I* > 2σ(*I*)
*R*_int_ = 0.039

#### Refinement

*R*[*F* ^2^ > 2σ(*F* ^2^)] = 0.046
*wR*(*F* ^2^) = 0.118
*S* = 1.02
9096 reflections
434 parameters
H-atom parameters constrained
Δρ_max_ = 0.25 e Å^−3^
Δρ_min_ = −0.33 e Å^−3^

### 

Data collection: *APEX2* (Bruker, 2004 ▶); cell refinement: *SAINT* (Bruker, 2004 ▶); data reduction: *SAINT*; program(s) used to solve structure: *SHELXS97* (Sheldrick, 2008 ▶); program(s) used to refine structure: *SHELXL97* (Sheldrick, 2008 ▶); molecular graphics: *PLATON* (Spek, 2009 ▶); software used to prepare material for publication: *SHELXL97*.

## Supplementary Material

Crystal structure: contains datablock(s) global, I. DOI: 10.1107/S1600536812007271/zj2058sup1.cif

Structure factors: contains datablock(s) I. DOI: 10.1107/S1600536812007271/zj2058Isup2.hkl

Additional supplementary materials:  crystallographic information; 3D view; checkCIF report

## Figures and Tables

**Table 1:** Hydrogen-bond geometry (Å, °) *Cg*1, *Cg*2 and *Cg*3 are the centroids of the rings C71–C76, C36–C41 and C32–C37, respectively.

*D*—H⋯*A*	*D*—H	H⋯*A*	*D*⋯*A*	*D*—H⋯*A*
C6—H6*B*⋯O2	0.97	2.46	2.956 (2)	111
C7—H7⋯O1	0.98	2.37	2.8012 (18)	106
C10—H10*A*⋯O2	0.97	2.56	3.188 (2)	123
C31—H31⋯O1	0.93	2.33	2.7296 (19)	105
C77—H77⋯O1	0.93	2.51	3.166 (2)	128
C9—H9*B*⋯O2^i^	0.97	2.50	3.357 (2)	147
C34—H34⋯O1^ii^	0.93	2.58	3.468 (2)	159
C22—H22⋯*Cg*1^iii^	0.93	2.77	3.483 (2)	134
C41—H41⋯*Cg*1^iv^	0.93	2.71	3.537 (3)	149
C74—H74⋯*Cg*2^v^	0.93	2.95	3.810 (2)	154
C80—H80⋯*Cg*3^v^	0.93	2.60	3.494 (3)	161

Symmetry codes: (i) 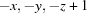; (ii) 
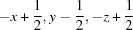
; (iii) 
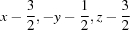
; (iv) 
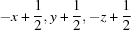
; (v) 
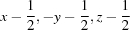
.

**Fig. 1.**
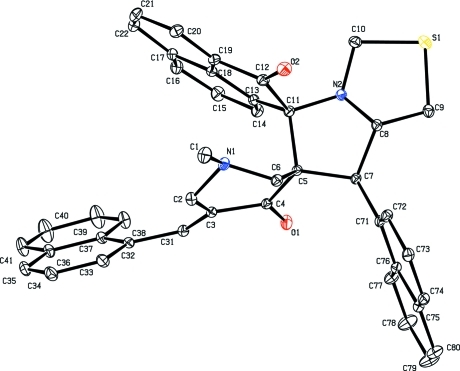
The molecular structure of (I), showing 10% probability displacement ellipsoids and the atom-numbering scheme. H atoms that do not take part in the H-bonding and some carbon atoms are omitted for clarity.

**Fig. 2.**
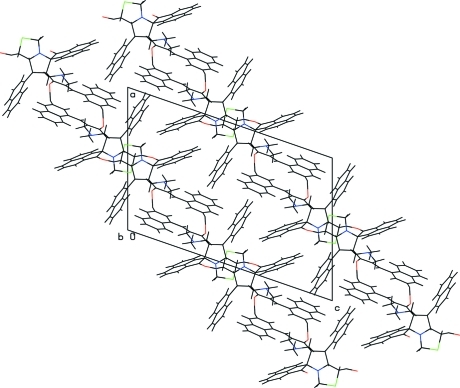
The partial packing diagram of the molecule.

## supplementary crystallographic information

### Comment

Spiro-compounds represent an important class of naturally occurring substances, which in many cases exhibit important biological properties (Kobayashi *et al.*, 1991; James *et al.*, 1991). 1,3-Dipolar cycloaddition reactions are widely used for the construction of spiro-compounds (Caramella & Grunanger, 1984).

In the title compound (Fig 1),*C*_43_H_34_N_2_O_2_S, the six-membered piperidine ring adopts half chair conformation with atoms N1 and C5 deviating by 0.600 (1) and 0.508 (1) Å, respectively, from the least-squares plane defined by atoms C2/C3/C4/C6. In the pyrrolo thiazole ring, the pyrrole ring is in the envelope conformation, and thiazole ring is in twisted envelope conformation with C8 atoms at the flap in both of these envelopes. The twisted envelope conformation of the thiazole may be due to the intramolecular C10—H10B···O2 interaction (Table 1). Similarly the orientation of the 4-methoxyphenyl substituent with respect to the attached piperidine ring may be influenced by the intermolecular C34—H34···O1 interaction (Table 1). The dihedral angle between the naphthyl rings are 83.7 (1)° and these rings are making angles of 51.5 (1)° and 35.2 (1)° with the acenaphthene group.

The C9—H9···O2 hydrogen bond connect two centrosymmetrically related molecules and generate the graph set motif *R*_2_^2^(14) (Fig. 2) Bernstein *et al.*, 1995). These dimers are connected into a zigzag chain by C34—H34···O1 hydrogen bonds (Fig. 2). In addition, there are four weak C—H···π interactions, *viz*., C22—H22···*Cg*1^iii^, C41—H41···*Cg*1^iv^ C74—H74···*Cg*2^v^ and C80—H80···*Cg*3^vi^ (*Cg*1,*Cg*2 and *Cg*3 are the centroids of the rings C71—C76,C36—C41 and C32—C37 respectively; symmetry codes are given in Table 1) are observed.

### Experimental

A mixture of 1-methyl-3,5-bis[(*E*)-naphthylmethylidene]tetrahydro-4(1*H*)-pyridinone (1 mmol), acenaphthenequinone (0.182 g, 1 mmol) and 1,3-thiazolane-4-carboxylic acid (0.133 g, 1 mmol) was dissolved in methanol (10 ml) and refluxed for 30 min. After completion of the reaction as evident from TLC, the mixture was poured into water (50 ml), the precipitated solid was filtered and washed with water (100 ml) to obtain pure product as pale yellow solid. The product was dissolved in 5 ml of ethyl acetate. The mixture was heated on a water bath to boiling and filtered to a beaker. The solution was kept aside undisturbed for crystallization *via* slow evaporation of the solvent. Fine crystals of the compound appeared as the solvent evaporated. Then the solvent was decanted and the crystals were washed with cold ethyl acetate to obtain suitable crystals for the X-ray analysis. Melting point:237 °C,Yield: 90%

### Refinement

H atoms were placed at calculated positions and allowed to ride on their carrier atoms with C—H = 0.93–0.98 Å. *U*_iso_ = 1.2*U*_eq_(C) for CH_2_ and CH groups and *U*_iso_ = 1.5*U*_eq_(C) for CH_3_ group.

### Crystal data

**Table:**

C_43_H_34_N_2_O_2_S	*F*(000) = 1352
*M**_r_* = 642.78	*D*_x_ = 1.291 Mg m^−^^3^
Monoclinic, *P*2_1_/*n*	Mo *K*α radiation, λ = 0.71073 Å
Hall symbol: -P 2yn	Cell parameters from 2000 reflections
*a* = 15.8335 (4) Å	θ = 2–31°
*b* = 9.2147 (2) Å	µ = 0.14 mm^−^^1^
*c* = 23.9951 (5) Å	*T* = 293 K
β = 109.103 (1)°	Block, colourless
*V* = 3308.12 (13) Å^3^	0.21 × 0.17 × 0.12 mm
*Z* = 4	

### Data collection

**Table:**

Bruker Kappa APEXII diffractometer	9096 independent reflections
Radiation source: fine-focus sealed tube	5614 reflections with *I* > 2σ(*I*)
Graphite monochromator	*R*_int_ = 0.039
Detector resolution: 0 pixels mm^-1^	θ_max_ = 29.4°, θ_min_ = 2.4°
ω and φ scans	*h* = −21→21
Absorption correction: multi-scan (*SADABS*; Sheldrick, 1996)	*k* = −11→12
*T*_min_ = 0.967, *T*_max_ = 0.974	*l* = −33→30
40746 measured reflections	

### Refinement

**Table:**

Refinement on *F*^2^	Secondary atom site location: difference Fourier map
Least-squares matrix: full	Hydrogen site location: inferred from neighbouring sites
*R*[*F*^2^ > 2σ(*F*^2^)] = 0.046	H-atom parameters constrained
*wR*(*F*^2^) = 0.118	*w* = 1/[σ^2^(*F*_o_^2^) + (0.0428*P*)^2^ + 0.8672*P*] where *P* = (*F*_o_^2^ + 2*F*_c_^2^)/3
*S* = 1.02	(Δ/σ)_max_ = 0.004
9096 reflections	Δρ_max_ = 0.25 e Å^−^^3^
434 parameters	Δρ_min_ = −0.33 e Å^−^^3^
0 restraints	Extinction correction: *SHELXL97* (Sheldrick, 2008), Fc^*^=kFc[1+0.001xFc^2^λ^3^/sin(2θ)]^-1/4^
Primary atom site location: structure-invariant direct methods	Extinction coefficient: 0.0011 (3)

### Special details

**Table:**

Geometry. All e.s.d.'s (except the e.s.d. in the dihedral angle between two l.s. planes) are estimated using the full covariance matrix. The cell e.s.d.'s are taken into account individually in the estimation of e.s.d.'s in distances, angles and torsion angles; correlations between e.s.d.'s in cell parameters are only used when they are defined by crystal symmetry. An approximate (isotropic) treatment of cell e.s.d.'s is used for estimating e.s.d.'s involving l.s. planes.
Refinement. Refinement of *F*^2^ against ALL reflections. The weighted *R*-factor *wR* and goodness of fit *S* are based on *F*^2^, conventional *R*-factors *R* are based on *F*, with *F* set to zero for negative *F*^2^. The threshold expression of *F*^2^ > σ(*F*^2^) is used only for calculating *R*-factors(gt) *etc*. and is not relevant to the choice of reflections for refinement. *R*-factors based on *F*^2^ are statistically about twice as large as those based on *F*, and *R*- factors based on ALL data will be even larger.

### Fractional atomic coordinates and isotropic or equivalent isotropic displacement parameters (Å^2^)

**Table:**

	*x*	*y*	*z*	*U*_iso_*/*U*_eq_	
C1	0.06539 (14)	−0.11977 (19)	0.30926 (8)	0.0598 (5)	
H1A	0.0355	−0.1317	0.2677	0.090*	
H1B	0.0308	−0.1650	0.3307	0.090*	
H1C	0.1234	−0.1641	0.3200	0.090*	
C2	0.12010 (11)	0.10877 (18)	0.28779 (7)	0.0445 (4)	
H2A	0.0836	0.1017	0.2466	0.053*	
H2B	0.1764	0.0604	0.2924	0.053*	
C3	0.13799 (9)	0.26601 (17)	0.30380 (6)	0.0361 (3)	
C4	0.13712 (10)	0.31838 (17)	0.36271 (6)	0.0355 (3)	
C5	0.09621 (9)	0.21822 (15)	0.39764 (6)	0.0316 (3)	
C6	0.11873 (10)	0.06270 (17)	0.38586 (6)	0.0387 (4)	
H6A	0.1829	0.0511	0.3961	0.046*	
H6B	0.0974	−0.0046	0.4094	0.046*	
C7	0.12624 (9)	0.26024 (16)	0.46452 (6)	0.0328 (3)	
H7	0.1335	0.3659	0.4669	0.039*	
C8	0.04289 (10)	0.22444 (16)	0.48068 (6)	0.0340 (3)	
H8	0.0351	0.1193	0.4826	0.041*	
C9	0.02946 (11)	0.29904 (18)	0.53306 (7)	0.0418 (4)	
H9A	0.0545	0.3961	0.5379	0.050*	
H9B	0.0571	0.2443	0.5689	0.050*	
C10	−0.11269 (11)	0.2781 (2)	0.43641 (7)	0.0463 (4)	
H10A	−0.1398	0.1841	0.4238	0.056*	
H10B	−0.1518	0.3531	0.4135	0.056*	
C11	−0.01036 (10)	0.24045 (16)	0.37577 (6)	0.0336 (3)	
C12	−0.06233 (10)	0.09732 (17)	0.34865 (7)	0.0400 (4)	
C13	−0.04961 (10)	0.34820 (17)	0.32593 (6)	0.0348 (3)	
C14	−0.04068 (11)	0.49462 (18)	0.32288 (7)	0.0432 (4)	
H14	−0.0043	0.5462	0.3551	0.052*	
C15	−0.08785 (12)	0.5669 (2)	0.26973 (8)	0.0527 (4)	
H15	−0.0816	0.6668	0.2676	0.063*	
C16	−0.14216 (12)	0.4952 (2)	0.22155 (7)	0.0538 (5)	
H16	−0.1712	0.5464	0.1872	0.065*	
C17	−0.15441 (11)	0.3445 (2)	0.22348 (7)	0.0458 (4)	
C18	−0.10720 (10)	0.27518 (18)	0.27658 (6)	0.0376 (4)	
C19	−0.11592 (10)	0.12690 (18)	0.28658 (7)	0.0418 (4)	
C20	−0.17101 (12)	0.0427 (2)	0.24284 (8)	0.0563 (5)	
H20	−0.1777	−0.0559	0.2486	0.068*	
C21	−0.21739 (13)	0.1100 (3)	0.18862 (8)	0.0671 (6)	
H21	−0.2538	0.0533	0.1581	0.081*	
C22	−0.21098 (12)	0.2544 (3)	0.17914 (8)	0.0603 (5)	
H22	−0.2442	0.2945	0.1430	0.072*	
C31	0.15384 (10)	0.36718 (19)	0.26847 (7)	0.0401 (4)	
H31	0.1635	0.4603	0.2842	0.048*	
C32	0.15823 (10)	0.35283 (18)	0.20865 (7)	0.0394 (4)	
C33	0.19016 (11)	0.2299 (2)	0.18976 (8)	0.0503 (4)	
H33	0.2078	0.1517	0.2154	0.060*	
C34	0.19698 (13)	0.2187 (2)	0.13315 (8)	0.0577 (5)	
H34	0.2189	0.1341	0.1218	0.069*	
C35	0.17160 (13)	0.3312 (2)	0.09517 (8)	0.0558 (5)	
H35	0.1757	0.3227	0.0575	0.067*	
C36	0.13910 (12)	0.4606 (2)	0.11151 (7)	0.0485 (4)	
C37	0.13197 (10)	0.47313 (18)	0.16895 (7)	0.0412 (4)	
C38	0.09868 (14)	0.6037 (2)	0.18350 (8)	0.0591 (5)	
H38	0.0917	0.6135	0.2203	0.071*	
C39	0.0765 (2)	0.7162 (2)	0.14472 (10)	0.0892 (8)	
H39	0.0554	0.8023	0.1555	0.107*	
C40	0.0850 (2)	0.7035 (3)	0.08869 (11)	0.0950 (9)	
H40	0.0703	0.7813	0.0626	0.114*	
C41	0.11441 (16)	0.5790 (3)	0.07265 (9)	0.0719 (6)	
H41	0.1186	0.5709	0.0350	0.086*	
C71	0.21277 (10)	0.19473 (16)	0.50429 (6)	0.0339 (3)	
C72	0.21646 (11)	0.05172 (17)	0.52050 (7)	0.0412 (4)	
H72	0.1648	−0.0040	0.5071	0.049*	
C73	0.29563 (11)	−0.01364 (19)	0.55661 (7)	0.0446 (4)	
H73	0.2960	−0.1117	0.5659	0.053*	
C74	0.37136 (11)	0.0655 (2)	0.57799 (7)	0.0454 (4)	
H74	0.4235	0.0215	0.6019	0.054*	
C75	0.37156 (10)	0.21369 (19)	0.56426 (7)	0.0428 (4)	
C76	0.29224 (10)	0.28108 (17)	0.52671 (6)	0.0373 (3)	
C77	0.29689 (12)	0.43031 (19)	0.51390 (8)	0.0527 (5)	
H77	0.2461	0.4774	0.4898	0.063*	
C78	0.37456 (14)	0.5059 (2)	0.53637 (10)	0.0768 (7)	
H78	0.3761	0.6037	0.5273	0.092*	
C79	0.45174 (14)	0.4386 (3)	0.57284 (11)	0.0831 (7)	
H79	0.5044	0.4915	0.5877	0.100*	
C80	0.45009 (12)	0.2973 (2)	0.58659 (9)	0.0640 (5)	
H80	0.5018	0.2539	0.6113	0.077*	
N1	0.07515 (9)	0.03429 (14)	0.32345 (6)	0.0406 (3)	
N2	−0.02549 (8)	0.28666 (14)	0.42998 (5)	0.0355 (3)	
O1	0.16719 (8)	0.43621 (13)	0.38166 (5)	0.0524 (3)	
O2	−0.06698 (8)	−0.00873 (13)	0.37765 (5)	0.0528 (3)	
S1	−0.09149 (3)	0.30508 (5)	0.51535 (2)	0.05001 (13)	

### Atomic displacement parameters (Å^2^)

**Table:**

	*U*^11^	*U*^22^	*U*^33^	*U*^12^	*U*^13^	*U*^23^
C1	0.0758 (13)	0.0410 (10)	0.0597 (12)	−0.0077 (9)	0.0183 (10)	−0.0120 (9)
C2	0.0494 (10)	0.0486 (10)	0.0363 (8)	0.0009 (8)	0.0151 (7)	−0.0021 (7)
C3	0.0317 (8)	0.0452 (9)	0.0309 (7)	−0.0024 (6)	0.0094 (6)	0.0010 (7)
C4	0.0328 (7)	0.0400 (9)	0.0319 (7)	−0.0065 (7)	0.0082 (6)	0.0020 (7)
C5	0.0313 (7)	0.0337 (8)	0.0282 (7)	−0.0045 (6)	0.0075 (6)	0.0024 (6)
C6	0.0396 (8)	0.0373 (9)	0.0368 (8)	−0.0019 (7)	0.0091 (7)	0.0025 (7)
C7	0.0350 (8)	0.0325 (8)	0.0290 (7)	−0.0054 (6)	0.0081 (6)	0.0026 (6)
C8	0.0389 (8)	0.0321 (8)	0.0305 (7)	−0.0018 (6)	0.0107 (6)	0.0060 (6)
C9	0.0500 (9)	0.0417 (9)	0.0352 (8)	−0.0021 (7)	0.0159 (7)	0.0048 (7)
C10	0.0389 (9)	0.0563 (11)	0.0453 (9)	0.0013 (8)	0.0161 (7)	0.0101 (8)
C11	0.0341 (8)	0.0343 (8)	0.0306 (7)	−0.0051 (6)	0.0083 (6)	0.0034 (6)
C12	0.0385 (8)	0.0372 (9)	0.0444 (9)	−0.0082 (7)	0.0135 (7)	0.0019 (7)
C13	0.0338 (8)	0.0401 (9)	0.0304 (7)	−0.0022 (6)	0.0104 (6)	0.0048 (6)
C14	0.0479 (9)	0.0401 (9)	0.0393 (9)	−0.0035 (7)	0.0114 (7)	0.0036 (7)
C15	0.0625 (11)	0.0447 (10)	0.0504 (10)	0.0018 (9)	0.0177 (9)	0.0156 (8)
C16	0.0550 (11)	0.0661 (13)	0.0382 (9)	0.0064 (9)	0.0123 (8)	0.0201 (9)
C17	0.0385 (9)	0.0669 (12)	0.0314 (8)	−0.0006 (8)	0.0108 (7)	0.0057 (8)
C18	0.0316 (8)	0.0488 (10)	0.0326 (8)	−0.0045 (7)	0.0107 (6)	0.0017 (7)
C19	0.0357 (8)	0.0481 (10)	0.0409 (9)	−0.0104 (7)	0.0113 (7)	−0.0031 (7)
C20	0.0509 (10)	0.0592 (12)	0.0560 (11)	−0.0188 (9)	0.0135 (9)	−0.0114 (9)
C21	0.0551 (12)	0.0938 (17)	0.0442 (11)	−0.0255 (11)	0.0051 (9)	−0.0185 (11)
C22	0.0491 (11)	0.0914 (16)	0.0348 (9)	−0.0087 (10)	0.0061 (8)	−0.0002 (10)
C31	0.0379 (8)	0.0479 (10)	0.0341 (8)	−0.0027 (7)	0.0112 (7)	0.0014 (7)
C32	0.0331 (8)	0.0528 (10)	0.0333 (8)	−0.0012 (7)	0.0121 (6)	0.0034 (7)
C33	0.0481 (10)	0.0607 (12)	0.0464 (10)	0.0129 (8)	0.0216 (8)	0.0118 (9)
C34	0.0585 (11)	0.0667 (13)	0.0562 (11)	0.0144 (10)	0.0302 (9)	0.0000 (10)
C35	0.0622 (11)	0.0734 (13)	0.0406 (9)	0.0054 (10)	0.0289 (9)	−0.0003 (9)
C36	0.0522 (10)	0.0606 (11)	0.0377 (9)	0.0001 (8)	0.0217 (8)	0.0068 (8)
C37	0.0417 (9)	0.0493 (10)	0.0355 (8)	−0.0023 (7)	0.0167 (7)	0.0033 (7)
C38	0.0862 (14)	0.0540 (12)	0.0448 (10)	0.0069 (10)	0.0321 (10)	0.0041 (9)
C39	0.156 (2)	0.0573 (14)	0.0704 (15)	0.0315 (15)	0.0585 (16)	0.0176 (11)
C40	0.162 (3)	0.0693 (16)	0.0706 (15)	0.0333 (16)	0.0617 (17)	0.0326 (13)
C41	0.1063 (18)	0.0751 (15)	0.0468 (11)	0.0134 (13)	0.0421 (12)	0.0171 (10)
C71	0.0374 (8)	0.0370 (8)	0.0260 (7)	−0.0032 (6)	0.0085 (6)	0.0022 (6)
C72	0.0427 (9)	0.0424 (9)	0.0347 (8)	−0.0050 (7)	0.0075 (7)	0.0065 (7)
C73	0.0511 (10)	0.0431 (9)	0.0379 (8)	0.0044 (8)	0.0124 (7)	0.0093 (7)
C74	0.0397 (9)	0.0586 (11)	0.0347 (8)	0.0089 (8)	0.0079 (7)	0.0048 (8)
C75	0.0384 (9)	0.0533 (11)	0.0347 (8)	−0.0016 (7)	0.0094 (7)	−0.0043 (7)
C76	0.0380 (8)	0.0425 (9)	0.0305 (7)	−0.0042 (7)	0.0099 (6)	−0.0043 (6)
C77	0.0483 (10)	0.0430 (10)	0.0568 (11)	−0.0091 (8)	0.0036 (8)	−0.0037 (8)
C78	0.0633 (13)	0.0512 (12)	0.0963 (17)	−0.0199 (10)	−0.0005 (12)	−0.0004 (11)
C79	0.0522 (12)	0.0700 (16)	0.1050 (19)	−0.0234 (11)	−0.0046 (12)	−0.0084 (13)
C80	0.0399 (10)	0.0703 (14)	0.0680 (13)	−0.0061 (9)	−0.0012 (9)	−0.0063 (11)
N1	0.0464 (8)	0.0357 (7)	0.0374 (7)	−0.0049 (6)	0.0107 (6)	−0.0031 (6)
N2	0.0344 (7)	0.0410 (7)	0.0315 (6)	−0.0017 (5)	0.0114 (5)	0.0063 (5)
O1	0.0703 (8)	0.0503 (7)	0.0391 (6)	−0.0287 (6)	0.0213 (6)	−0.0052 (5)
O2	0.0583 (7)	0.0406 (7)	0.0588 (7)	−0.0131 (6)	0.0182 (6)	0.0090 (6)
S1	0.0556 (3)	0.0512 (3)	0.0522 (3)	−0.0008 (2)	0.0300 (2)	0.0002 (2)

### Geometric parameters (Å, º)

**Table:**

C1—N1	1.456 (2)	C18—C19	1.402 (2)
C1—H1A	0.9600	C19—C20	1.366 (2)
C1—H1B	0.9600	C20—C21	1.411 (3)
C1—H1C	0.9600	C20—H20	0.9300
C2—N1	1.452 (2)	C21—C22	1.359 (3)
C2—C3	1.502 (2)	C21—H21	0.9300
C2—H2A	0.9700	C22—H22	0.9300
C2—H2B	0.9700	C31—C32	1.465 (2)
C3—C31	1.338 (2)	C31—H31	0.9300
C3—C4	1.498 (2)	C32—C33	1.376 (2)
C4—O1	1.2127 (18)	C32—C37	1.432 (2)
C4—C5	1.526 (2)	C33—C34	1.401 (2)
C5—C6	1.525 (2)	C33—H33	0.9300
C5—C7	1.5658 (19)	C34—C35	1.352 (3)
C5—C11	1.608 (2)	C34—H34	0.9300
C6—N1	1.4530 (19)	C35—C36	1.404 (3)
C6—H6A	0.9700	C35—H35	0.9300
C6—H6B	0.9700	C36—C41	1.406 (3)
C7—C71	1.516 (2)	C36—C37	1.424 (2)
C7—C8	1.528 (2)	C37—C38	1.403 (2)
C7—H7	0.9800	C38—C39	1.360 (3)
C8—N2	1.4555 (18)	C38—H38	0.9300
C8—C9	1.508 (2)	C39—C40	1.400 (3)
C8—H8	0.9800	C39—H39	0.9300
C9—S1	1.8216 (17)	C40—C41	1.341 (3)
C9—H9A	0.9700	C40—H40	0.9300
C9—H9B	0.9700	C41—H41	0.9300
C10—N2	1.441 (2)	C71—C72	1.370 (2)
C10—S1	1.8292 (17)	C71—C76	1.436 (2)
C10—H10A	0.9700	C72—C73	1.405 (2)
C10—H10B	0.9700	C72—H72	0.9300
C11—N2	1.4611 (19)	C73—C74	1.353 (2)
C11—C13	1.522 (2)	C73—H73	0.9300
C11—C12	1.578 (2)	C74—C75	1.405 (2)
C12—O2	1.2158 (18)	C74—H74	0.9300
C12—C19	1.478 (2)	C75—C80	1.411 (2)
C13—C14	1.361 (2)	C75—C76	1.426 (2)
C13—C18	1.407 (2)	C76—C77	1.416 (2)
C14—C15	1.416 (2)	C77—C78	1.362 (2)
C14—H14	0.9300	C77—H77	0.9300
C15—C16	1.364 (2)	C78—C79	1.394 (3)
C15—H15	0.9300	C78—H78	0.9300
C16—C17	1.405 (3)	C79—C80	1.346 (3)
C16—H16	0.9300	C79—H79	0.9300
C17—C18	1.402 (2)	C80—H80	0.9300
C17—C22	1.414 (2)		
			
N1—C1—H1A	109.5	C20—C19—C12	132.93 (16)
N1—C1—H1B	109.5	C18—C19—C12	107.21 (13)
H1A—C1—H1B	109.5	C19—C20—C21	117.87 (18)
N1—C1—H1C	109.5	C19—C20—H20	121.1
H1A—C1—H1C	109.5	C21—C20—H20	121.1
H1B—C1—H1C	109.5	C22—C21—C20	122.67 (17)
N1—C2—C3	113.14 (13)	C22—C21—H21	118.7
N1—C2—H2A	109.0	C20—C21—H21	118.7
C3—C2—H2A	109.0	C21—C22—C17	120.82 (17)
N1—C2—H2B	109.0	C21—C22—H22	119.6
C3—C2—H2B	109.0	C17—C22—H22	119.6
H2A—C2—H2B	107.8	C3—C31—C32	129.54 (16)
C31—C3—C4	115.79 (14)	C3—C31—H31	115.2
C31—C3—C2	124.67 (14)	C32—C31—H31	115.2
C4—C3—C2	119.53 (13)	C33—C32—C37	118.43 (14)
O1—C4—C3	121.23 (14)	C33—C32—C31	122.49 (15)
O1—C4—C5	121.37 (13)	C37—C32—C31	119.03 (15)
C3—C4—C5	117.39 (13)	C32—C33—C34	122.23 (16)
C6—C5—C4	107.42 (12)	C32—C33—H33	118.9
C6—C5—C7	114.28 (12)	C34—C33—H33	118.9
C4—C5—C7	111.85 (11)	C35—C34—C33	119.78 (17)
C6—C5—C11	110.07 (11)	C35—C34—H34	120.1
C4—C5—C11	109.42 (11)	C33—C34—H34	120.1
C7—C5—C11	103.72 (11)	C34—C35—C36	121.20 (16)
N1—C6—C5	107.41 (12)	C34—C35—H35	119.4
N1—C6—H6A	110.2	C36—C35—H35	119.4
C5—C6—H6A	110.2	C35—C36—C41	121.46 (16)
N1—C6—H6B	110.2	C35—C36—C37	119.46 (16)
C5—C6—H6B	110.2	C41—C36—C37	119.07 (17)
H6A—C6—H6B	108.5	C38—C37—C36	117.78 (15)
C71—C7—C8	115.60 (12)	C38—C37—C32	123.32 (15)
C71—C7—C5	117.10 (12)	C36—C37—C32	118.90 (15)
C8—C7—C5	102.05 (11)	C39—C38—C37	121.17 (17)
C71—C7—H7	107.2	C39—C38—H38	119.4
C8—C7—H7	107.2	C37—C38—H38	119.4
C5—C7—H7	107.2	C38—C39—C40	120.6 (2)
N2—C8—C9	104.35 (12)	C38—C39—H39	119.7
N2—C8—C7	99.84 (11)	C40—C39—H39	119.7
C9—C8—C7	118.83 (13)	C41—C40—C39	120.0 (2)
N2—C8—H8	111.0	C41—C40—H40	120.0
C9—C8—H8	111.0	C39—C40—H40	120.0
C7—C8—H8	111.0	C40—C41—C36	121.42 (18)
C8—C9—S1	104.11 (10)	C40—C41—H41	119.3
C8—C9—H9A	110.9	C36—C41—H41	119.3
S1—C9—H9A	110.9	C72—C71—C76	118.42 (14)
C8—C9—H9B	110.9	C72—C71—C7	120.52 (13)
S1—C9—H9B	110.9	C76—C71—C7	121.06 (13)
H9A—C9—H9B	109.0	C71—C72—C73	122.28 (15)
N2—C10—S1	104.23 (10)	C71—C72—H72	118.9
N2—C10—H10A	110.9	C73—C72—H72	118.9
S1—C10—H10A	110.9	C74—C73—C72	120.28 (16)
N2—C10—H10B	110.9	C74—C73—H73	119.9
S1—C10—H10B	110.9	C72—C73—H73	119.9
H10A—C10—H10B	108.9	C73—C74—C75	120.30 (15)
N2—C11—C13	111.45 (12)	C73—C74—H74	119.9
N2—C11—C12	113.30 (12)	C75—C74—H74	119.9
C13—C11—C12	101.39 (11)	C74—C75—C80	120.74 (16)
N2—C11—C5	101.94 (11)	C74—C75—C76	120.14 (15)
C13—C11—C5	117.04 (12)	C80—C75—C76	119.11 (17)
C12—C11—C5	112.22 (12)	C77—C76—C75	117.54 (14)
O2—C12—C19	127.05 (14)	C77—C76—C71	123.92 (14)
O2—C12—C11	123.84 (14)	C75—C76—C71	118.55 (14)
C19—C12—C11	108.14 (12)	C78—C77—C76	120.99 (18)
C14—C13—C18	118.62 (14)	C78—C77—H77	119.5
C14—C13—C11	131.77 (14)	C76—C77—H77	119.5
C18—C13—C11	109.57 (13)	C77—C78—C79	120.9 (2)
C13—C14—C15	118.75 (15)	C77—C78—H78	119.5
C13—C14—H14	120.6	C79—C78—H78	119.5
C15—C14—H14	120.6	C80—C79—C78	120.05 (19)
C16—C15—C14	122.43 (17)	C80—C79—H79	120.0
C16—C15—H15	118.8	C78—C79—H79	120.0
C14—C15—H15	118.8	C79—C80—C75	121.39 (18)
C15—C16—C17	120.39 (15)	C79—C80—H80	119.3
C15—C16—H16	119.8	C75—C80—H80	119.3
C17—C16—H16	119.8	C2—N1—C6	111.30 (12)
C18—C17—C16	116.22 (15)	C2—N1—C1	110.98 (14)
C18—C17—C22	115.74 (17)	C6—N1—C1	113.26 (13)
C16—C17—C22	128.01 (16)	C10—N2—C8	111.45 (12)
C19—C18—C17	123.12 (15)	C10—N2—C11	121.43 (12)
C19—C18—C13	113.27 (13)	C8—N2—C11	109.60 (11)
C17—C18—C13	123.56 (15)	C9—S1—C10	93.45 (7)
C20—C19—C18	119.75 (16)		
			
N1—C2—C3—C31	−159.63 (15)	C19—C20—C21—C22	−1.7 (3)
N1—C2—C3—C4	19.3 (2)	C20—C21—C22—C17	1.9 (3)
C31—C3—C4—O1	−14.9 (2)	C18—C17—C22—C21	−0.3 (3)
C2—C3—C4—O1	166.07 (15)	C16—C17—C22—C21	−177.92 (19)
C31—C3—C4—C5	164.54 (13)	C4—C3—C31—C32	−179.29 (14)
C2—C3—C4—C5	−14.5 (2)	C2—C3—C31—C32	−0.3 (3)
O1—C4—C5—C6	−145.08 (15)	C3—C31—C32—C33	−33.7 (3)
C3—C4—C5—C6	35.45 (16)	C3—C31—C32—C37	149.05 (17)
O1—C4—C5—C7	−18.9 (2)	C37—C32—C33—C34	−0.5 (3)
C3—C4—C5—C7	161.62 (12)	C31—C32—C33—C34	−177.82 (16)
O1—C4—C5—C11	95.45 (16)	C32—C33—C34—C35	−0.1 (3)
C3—C4—C5—C11	−84.02 (15)	C33—C34—C35—C36	0.6 (3)
C4—C5—C6—N1	−63.73 (14)	C34—C35—C36—C41	178.6 (2)
C7—C5—C6—N1	171.56 (12)	C34—C35—C36—C37	−0.6 (3)
C11—C5—C6—N1	55.33 (15)	C35—C36—C37—C38	−179.61 (17)
C6—C5—C7—C71	35.22 (17)	C41—C36—C37—C38	1.2 (3)
C4—C5—C7—C71	−87.11 (16)	C35—C36—C37—C32	0.0 (2)
C11—C5—C7—C71	155.07 (12)	C41—C36—C37—C32	−179.23 (17)
C6—C5—C7—C8	−92.05 (14)	C33—C32—C37—C38	−179.90 (17)
C4—C5—C7—C8	145.62 (12)	C31—C32—C37—C38	−2.5 (2)
C11—C5—C7—C8	27.80 (13)	C33—C32—C37—C36	0.6 (2)
C71—C7—C8—N2	−172.66 (12)	C31—C32—C37—C36	177.95 (14)
C5—C7—C8—N2	−44.43 (13)	C36—C37—C38—C39	−1.9 (3)
C71—C7—C8—C9	74.84 (17)	C32—C37—C38—C39	178.5 (2)
C5—C7—C8—C9	−156.93 (13)	C37—C38—C39—C40	1.0 (4)
N2—C8—C9—S1	41.37 (13)	C38—C39—C40—C41	0.7 (5)
C7—C8—C9—S1	151.38 (11)	C39—C40—C41—C36	−1.5 (4)
C6—C5—C11—N2	121.79 (12)	C35—C36—C41—C40	−178.7 (2)
C4—C5—C11—N2	−120.38 (12)	C37—C36—C41—C40	0.5 (4)
C7—C5—C11—N2	−0.89 (13)	C8—C7—C71—C72	46.07 (19)
C6—C5—C11—C13	−116.36 (14)	C5—C7—C71—C72	−74.28 (18)
C4—C5—C11—C13	1.47 (17)	C8—C7—C71—C76	−132.81 (15)
C7—C5—C11—C13	120.96 (13)	C5—C7—C71—C76	106.84 (16)
C6—C5—C11—C12	0.25 (16)	C76—C71—C72—C73	−2.0 (2)
C4—C5—C11—C12	118.08 (13)	C7—C71—C72—C73	179.11 (14)
C7—C5—C11—C12	−122.42 (12)	C71—C72—C73—C74	1.7 (2)
N2—C11—C12—O2	−43.5 (2)	C72—C73—C74—C75	0.0 (2)
C13—C11—C12—O2	−162.99 (15)	C73—C74—C75—C80	179.66 (17)
C5—C11—C12—O2	71.33 (19)	C73—C74—C75—C76	−1.2 (2)
N2—C11—C12—C19	125.98 (14)	C74—C75—C76—C77	−179.24 (15)
C13—C11—C12—C19	6.44 (16)	C80—C75—C76—C77	−0.1 (2)
C5—C11—C12—C19	−119.24 (13)	C74—C75—C76—C71	0.9 (2)
N2—C11—C13—C14	51.2 (2)	C80—C75—C76—C71	−179.99 (16)
C12—C11—C13—C14	172.09 (17)	C72—C71—C76—C77	−179.19 (16)
C5—C11—C13—C14	−65.5 (2)	C7—C71—C76—C77	−0.3 (2)
N2—C11—C13—C18	−126.06 (13)	C72—C71—C76—C75	0.7 (2)
C12—C11—C13—C18	−5.22 (16)	C7—C71—C76—C75	179.60 (13)
C5—C11—C13—C18	117.18 (14)	C75—C76—C77—C78	0.5 (3)
C18—C13—C14—C15	−1.6 (2)	C71—C76—C77—C78	−179.65 (19)
C11—C13—C14—C15	−178.72 (16)	C76—C77—C78—C79	−0.2 (4)
C13—C14—C15—C16	0.3 (3)	C77—C78—C79—C80	−0.4 (4)
C14—C15—C16—C17	1.0 (3)	C78—C79—C80—C75	0.7 (4)
C15—C16—C17—C18	−0.9 (3)	C74—C75—C80—C79	178.6 (2)
C15—C16—C17—C22	176.71 (18)	C76—C75—C80—C79	−0.5 (3)
C16—C17—C18—C19	176.50 (16)	C3—C2—N1—C6	−48.62 (18)
C22—C17—C18—C19	−1.4 (2)	C3—C2—N1—C1	−175.73 (14)
C16—C17—C18—C13	−0.6 (2)	C5—C6—N1—C2	73.38 (16)
C22—C17—C18—C13	−178.43 (15)	C5—C6—N1—C1	−160.76 (14)
C14—C13—C18—C19	−175.49 (14)	S1—C10—N2—C8	35.34 (15)
C11—C13—C18—C19	2.22 (18)	S1—C10—N2—C11	166.82 (11)
C14—C13—C18—C17	1.8 (2)	C9—C8—N2—C10	−51.88 (16)
C11—C13—C18—C17	179.53 (14)	C7—C8—N2—C10	−175.23 (13)
C17—C18—C19—C20	1.5 (2)	C9—C8—N2—C11	170.85 (12)
C13—C18—C19—C20	178.87 (15)	C7—C8—N2—C11	47.51 (14)
C17—C18—C19—C12	−175.14 (15)	C13—C11—N2—C10	73.17 (17)
C13—C18—C19—C12	2.18 (18)	C12—C11—N2—C10	−40.42 (19)
O2—C12—C19—C20	−12.6 (3)	C5—C11—N2—C10	−161.21 (13)
C11—C12—C19—C20	178.41 (18)	C13—C11—N2—C8	−154.57 (12)
O2—C12—C19—C18	163.48 (17)	C12—C11—N2—C8	91.83 (14)
C11—C12—C19—C18	−5.51 (17)	C5—C11—N2—C8	−28.95 (14)
C18—C19—C20—C21	0.0 (3)	C8—C9—S1—C10	−19.62 (12)
C12—C19—C20—C21	175.70 (18)	N2—C10—S1—C9	−7.52 (12)

### Hydrogen-bond geometry (Å, º)

**Table:**

*D*—H···*A*	*D*—H	H···*A*	*D*···*A*	*D*—H···*A*
C6—H6*B*···O2	0.97	2.46	2.956 (2)	111
C7—H7···O1	0.98	2.37	2.8012 (18)	106
C10—H10*A*···O2	0.97	2.56	3.188 (2)	123
C31—H31···O1	0.93	2.33	2.7296 (19)	105
C77—H77···O1	0.93	2.51	3.166 (2)	128
C9—H9*B*···O2^i^	0.97	2.50	3.357 (2)	147
C34—H34···O1^ii^	0.93	2.58	3.468 (2)	159
C22—H22···*Cg*1^iii^	0.93	2.77	3.483 (2)	134
C41—H41···*Cg*1^iv^	0.93	2.71	3.537 (3)	149
C74—H74···*Cg*2^v^	0.93	2.95	3.810 (2)	154
C80—H80···*Cg*3^v^	0.93	2.60	3.494 (3)	161

Symmetry codes: (i) −*x*, −*y*, −*z*+1; (ii) −*x*+1/2, *y*−1/2, −*z*+1/2; (iii) *x*−3/2, −*y*−1/2, *z*−3/2; (iv) −*x*+1/2, *y*+1/2, −*z*+1/2; (v) *x*−1/2, −*y*−1/2, *z*−1/2.
